# Impact of SARS-CoV-2 Lockdown on the Preoperative Care Program of Patients Scheduled for Bariatric Surgery

**DOI:** 10.3390/nu14071488

**Published:** 2022-04-02

**Authors:** Luigi Schiavo, Pietro Calabrese, Silvana Mirella Aliberti, Salvatore Tramontano, Antonio Iannelli, Vincenzo Pilone

**Affiliations:** 1 Department of Medicine, Surgery, and Dentistry, “Scuola Medica Salernitana”, University of Salerno, Baronissi, 84081 Salerno, Italy; p.calabrese@unisa.it (P.C.); s.aliberti@unisa.it (S.M.A.); s.tramontano@unisa.it (S.T.); v.pilone@unisa.it (V.P.); 2 Center of Excellence of Bariatric Surgery of the Italian Society of Obesity Surgery and Metabolic Disease (SICOB), Unit of General and Emergency Surgery, University Hospital San Giovanni di Dio e Ruggi d’Aragona, Mercato San Severino, 84085 Salerno, Italy; 3 Digestive Unit, Archet 2 Hospital, University Hospital of Nice, F-06202 Nice, France; a.iannelli@nice-chu.fr; 4 Inserm, U1065, Team 8 “Hepatic Complications of Obesity”, F-06204 Nice, France; 5 Faculty of Medicine, University of Nice Sophia-Antipolis, F-06107 Nice, France

**Keywords:** obesity, bariatric surgery, preoperative care, COVID-19, micronutrient deficiencies

## Abstract

Objectives: To evaluate the effect of the SARS-CoV-2 lockdown on dietary habits, body weight, left hepatic lobe volume, use of micronutrient supplements, micronutrient status, frequency of physical activity, and evolution of comorbidities in patients undergoing preoperative care for BS. Materials and Methods: We prospectively evaluated the dietary habits (including use of micronutrient supplements and frequency of physical activity) of 36 patients who were candidates for BS from March to May 2020; 7-day food dietary records, body weight, left hepatic lobe volume by ultrasound, micronutrient status, and evolution of comorbidities were assessed. Results: All patients completed the study. Of the participants, 44.4% (16/36), 47.2% (17/36), and 27.8% (10/36) followed the preoperative indications for vegetables, fruits, and legumes, respectively, whereas over 50% did not. Furthermore, 30.6% (11/36) and 55.6% (20/36) of participants followed the prescribed recommendations for carbohydrates/sweets products and alcohol, respectively. A total of 61.1% (22/36) of participants experienced new foods and new culinary preparations. In addition, at the time of the study, we found that only 11.1% (4/36) were engaged in prescribed physical activity and only 36.1% (13/36) were taking prescribed micronutrient supplements. Compared to the initial weight, we observed an increased body weight and body mass index (+4.9%, *p* = 0.115; +1.89%, *p* = 0.0692, respectively), and no improvement in left hepatic lobe volume, micronutrient status, or comorbidities was recorded for any patient in the anamnesis. Conclusions: Lockdown determined by the SARS-CoV-2 pandemic has negatively affected the preoperative program of BS candidates, resulting in a postponement to the resumption of bariatric surgical activity.

## 1. Introduction

The current coronavirus disease 2019 (COVID-19) pandemic [1] is a challenge to daily clinical and surgical practice. Because of increasing morbidity and mortality in the northern region of Italy, a national lockdown was instituted on 9 March 2020 [2]. In particular, COVID-19 disease has been creating a fast and stressful public health emergency in Italy, and, as a consequence, the major priority was to reduce the surgical procedures to the bare necessity. As a consequence, all elective bariatric procedures were stopped in our university hospital. Bariatric surgery (BS) represents the most efficient and durable therapeutic means for the long-term treatment of severe obesity [3]. Nowadays, BS is performed laparoscopically in most cases. Patients who are candidates for BS frequently show an enlarged steatotic liver and increased intra-abdominal fat that may render the surgical procedure technically difficult [4,5]. Therefore, preoperative interventions to reduce liver volume and intra-abdominal fat before laparoscopic BS are often recommended [5,6]. Moreover, it is common in BS patients to have micronutrient deficiencies (MD) that may, in turn, be exacerbated by the bariatric procedure, resulting in dangerous postoperative complications [7,8]. Several diets have been proven to be effective in determining a preoperative weight loss associated with a reduction in liver volume. In this context, the ketogenic diet has recently been proposed as an attractive nutritional strategy for the treatment of obesity. The ketogenic diet is a very low-carb, high-fat diet consisting of 90% calories from fat and 10% from proteins and carbohydrates [9]. We have shown previously that a 4-week preoperative ketogenic micronutrient enriched diet (KMED) is safe and effective in reducing body weight (BW), left hepatic lobe volume, and correcting MD in patients with obesity scheduled for BS [10]. During the COVID-19 pandemic era, a large chapter of research on food intake started, and in particular, the “COVIDiet” study has been carried out in various countries, with the aim of describing the changes in the diet that followed the lockdown and the resulting effects on body weight. The vast majority of papers indicate an increase in body weight during lockdown [11].

This prospective pilot study aimed to assess the impact of the lockdown caused by SARS-CoV-2 on dietary habits, BW, left hepatic lobe volume, use of micronutrient supplements, micronutrient status, frequency of physical activity, and evolution of comorbidities in a cohort of patients undergoing preoperative care for BS.

## 2. Materials and Methods

### 2.1. Study Design and Characteristics of the Study Patients at Baseline

Between March and May 2020, we conducted a prospective study of a cohort of patients with obesity scheduled for BS at our University Hospital. The study included 36 subjects (24 females and 12 males) with a mean initial weight and body mass index (BMI) of 143.6 ± 23.6 kg and 50.1 ± 5.9 kg/m^2^, respectively. All patients fulfilled the criteria for surgical treatment for morbid obesity established by the International Federation for Surgery of Obesity. Exclusion criteria were as follows: serum creatinine level greater than 1.8 mg/dL; glutamic oxaloacetic transaminase or glutamic pyruvic transaminase [GOT and GPT, respectively]) levels less than three times the upper limit of normal. Because of the pandemic, health care providers can only use telemedicine to provide the patients with a prescription for a blood test to investigate micronutrient status. Patients were also instructed on how to fill the food diary record (FDR), and information on weight, comorbidity evolution, and physical activity were gathered.

### 2.2. Endpoints

The primary endpoint was the deviation of the patients’ diet from the preoperative recommendations. Secondary endpoints were changes in BW and left hepatic lobe volume, the evolution of comorbidities, frequency of physical activity, use of micronutrient supplements, and micronutrient status.

### 2.3. Development of the KMED

**Figure 1:**
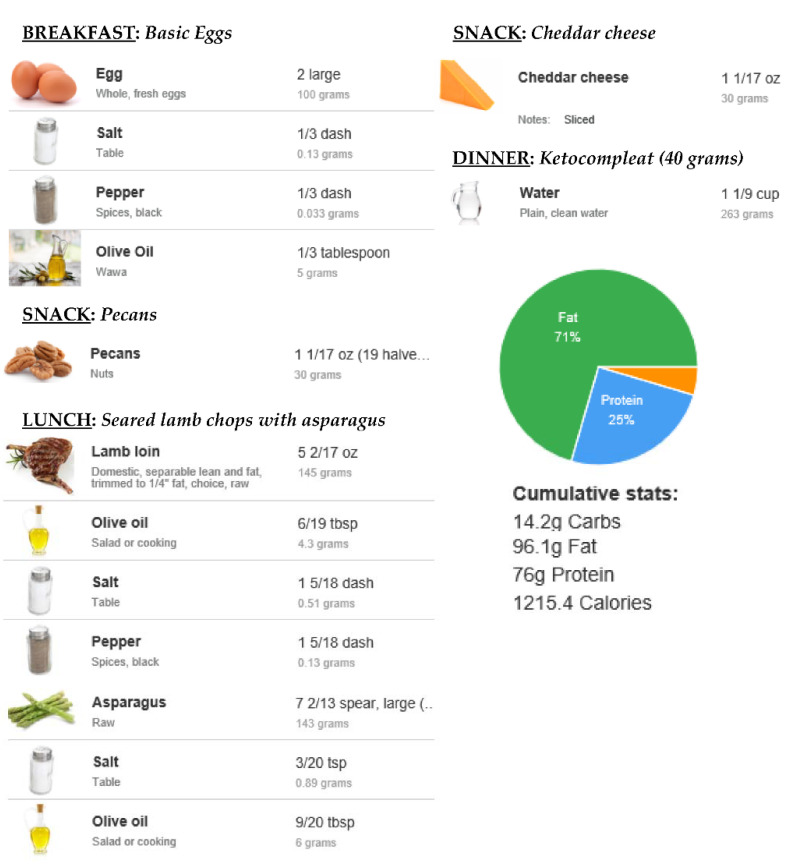
KMED daily plan example.

Before starting the KMED, participants were counseled individually about the dietetic protocol that they would be expected to follow for four weeks. To guarantee that all 36 of the included participants consumed a similar diet, we developed two KMED meal plans, plan 1 (from day 1 to day 14) and plan 2 (from day 15 to day 28), assigning a precise quantity to individual foods using a piece of free online software (https://www.eatthismuch.com, (accessed on 10 January 2020)). Each ketogenic food plan (from 1150 to 1250 Kcal/day) consisted of 25% proteins, 4% carbohydrates, and 71% fats. An example is reported in Figure 1, whereas the composition of the supplement (Ketocompleat, MVMedical Solutions, Serravalle, Repubblica San Marino) is reported in Table 1 [10,12].

### 2.4. Nutritional Counseling, FDR, and Nutrient Intake Assessment

FDR is a self-reported account of all foods and beverages, and dietary supplements consumed by a respondent over one or more days [13,14]. Typically, participants are asked to record foods and beverages that are consumed throughout the reporting day. All respondents were counseled individually via videocall by a trained bariatric nutritionist. The use and the significance of the FDR were explained. Each subject was then asked to complete an FDR for seven consecutive days to evaluate their dietary habits, and successively compare them with those of KMED recommendations. Participants were also asked to record dietary supplements consumed, and the frequency of physical activity. Furthermore, the evolution of preoperative comorbidities was also investigated.

### 2.5. Anthropometric Evaluation, Blood Tests, and Micronutrient Status of the Study Population

BW evaluation was done at baseline and repeated weekly by the same nutritionist (LS) during the 4-week follow-up. As shown in Table 2, blood tests included liver enzyme levels, kidney parameters, glycemic profile parameters, uric acid levels, ketonemia, iron, hemoglobin, and lipid profile. The GFR was calculated using the Modification of Diet in Renal Disease formula [15]. Furthermore, the assessment of micronutrient status included vitamin B12, vitamin D, vitamin C, vitamin A, vitamin E, folate, iron, zinc [16], magnesium, and selenium. All blood analyses were performed in an approved laboratory at baseline and repeated at 4-week follow-up and compared with accepted clinical cutoff values.

### 2.6. Left Hepatic Lobe Volume Measurement

Liver ultrasonography measurement was done at baseline and repeated at 4-week follow-up by a single trained radiologist using an M7 Diagnostic Ultrasound System (Mindray Medical International, Milano, Italy). In agreement with previous studies [5,17], the left hepatic lobe volume was calculated as follows: transversal and superoinferior axis, and the middle part of the anteroposterior axis (“thickness”), assuming that its shape was close to a half-rectangular parallelepiped.

### 2.7. Statistical Analysis

The BW and BMI of the patient pre- and post-KMED were directly compared by using the paired-sample t test for continuous variables (Graph Pad Software, San Diego, CA, USA). Any *p* value < 0.05 was considered statistically significant. Results on BW, frequency of physical activity, the evolution of comorbidities, use of micronutrient supplements, micronutrient status, and dietary habits at follow-up were analyzed using descriptive statistics. Heat map representations (MultiExperiment Viewer software, J. Craig Venter Institute, 4120 Capricorn Lane, La Jolla, CA 92037, USA) were used to depict the data of the deviance by the participants from the prescribed preoperative recommendations.

## 3. Results

### 3.1. Dietary Habits Assessment and Comparison with the Prescribed KMED

**Figure 2:**
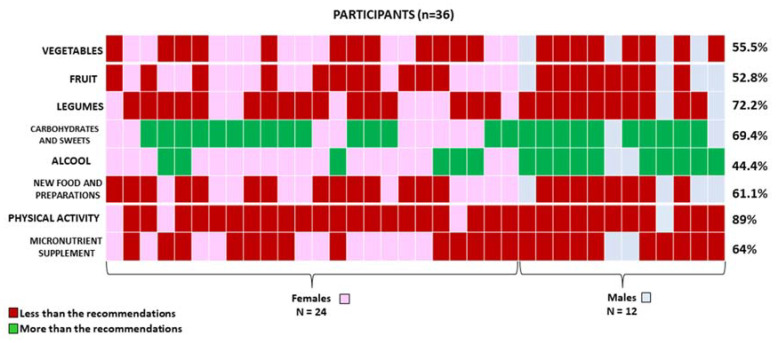
Heat map representation of participants who stated following less (red) or more (green) than the KMED preoperative recommendations.

As shown in Figure 2, only 44.5% (16/36), 47.2% (17/36), and 27.8% (10/36) of the participants followed the preoperative indications for main meal portions of vegetables, fruits, and legumes, respectively, whereas all the other participants (more than half) did not. Furthermore, we found that 30.6% (11/36) and 55.6% (20/36) of participants followed the prescribed recommendations for carbohydrates/sweet products and alcohol, respectively. Furthermore, 61.1% (22/36) of participants reported experiencing new foods and new culinary preparations.

### 3.2. Changes in BW and Left Hepatic Lobe Volume

As shown in Table 2, mean pre-KMED BW and BMI were 143.6 ± 23.6 kg and 50.1 ± 5.9 kg/m^2^, respectively, whereas mean BW and BMI at the time of follow-up were 151 ± 16.1 kg (*p* = 0.115) and 52.8 ± 6.5 kg/m^2^ (*p* = 0.0692), respectively. Therefore, at the time of follow-up, we did not record any weight loss (Table 2). Furthermore, as shown in Table 2, at the time of follow-up, the mean volume of the left hepatic lobe did not show any significant variation compared to the baseline (*p* = 0.0848).

### 3.3. Use of Micronutrient Supplements, Micronutrient Status, Frequency of Physical Activity, and Evolution of Comorbidities

As shown in Figure 2, only 36% (13/36) of participants were taking the prescribed micronutrient supplements at the time of the study. Furthermore, several micronutrient deficiencies were found before the pre-BS care program was started. In particular, pre-KMED vitamin B12, folate, vitamin D, iron, and zinc deficiencies were found in 41.7% (15/36), 38.9% (14/36), 61.1% (22/36), 33.3% (12/36), and 25% (9/36) of participants, respectively. At the time of follow-up, no amelioration was found (Table 2). Moreover, we found that only 11.1% (4/36) of participants were engaged in prescribed physical activity (1/4 and 3/4 of the 11.1% declared they were involved in bouts of moderate-to-vigorous physical activity at least two or three times a week, respectively) whereas the remaining participants (32/36) were not involved in any bouts of moderate-to-vigorous physical activity. Finally, pre-KMED T2D, hypertension, and dyslipidemia were present in 16.7% (6/36), 22.2% (8/36), and 22.2 (8/36) of participants, respectively. At the time of follow-up, no amelioration was found (Table 2). In addition, as shown in Table 2, we did not observe an improvement in the clinical status of the patients. After the 4-week KMED, the mean blood ketone levels were similar to the baseline, indicating a non-adherence in following the prescribed diet (Table 2).

## 4. Discussion

Herein, we show that the lockdown determined by the SARS-CoV-2 pandemic has negatively affected the preoperative program of patients with obesity who were scheduled for BS, resulting in a postponement of surgery until the resumption of surgical activity. The aim of the pre-BS program is not only in weight loss to simplify the surgical procedure but also to correct MD, and to improve IR and obesity-linked low-grade systemic inflammation [6,18,19]. Numerous studies have been shown to decrease BW, left hepatic lobe volume, and correct MD in patients with obesity scheduled for BS [10,20]. Herein, we report that in a cohort of patients undergoing preoperative care awaiting BS, no amelioration was found in terms of BW, left hepatic lobe volume, micronutrient status, or comorbidities. From a nutritional point of view, several diets have been proven to be effective in determining a preoperative weight loss associated with a reduction in liver volume. In this context, the ketogenic diet has recently been proposed as an attractive nutritional strategy for the treatment of obesity, even in patients scheduled for BS [10].

The ketogenic diet produces a fast and sensible weight loss associated with beneficial clinical marker changes, such as a significant reduction in serum glycated hemoglobin in patients suffering from diabetes type 2. On the contrary, it seems to produce a considerable increase in triglycerides and low-density lipoprotein cholesterol levels [21]. As a consequence, many nutritionists are hesitant to recommend the ketogenic diet, mostly in those subjects who are not in need of weight loss. Moreover, further and larger randomized clinical trials are needed to assess the potential long-term consequences of the ketogenic diet.

From a surgical point of view, an enlarged liver associated with increased intra-abdominal fat represents two major contributing factors of technical complexity that may be faced in executing any BS procedure [22,23,24,25,26].

In fact, in these patients, the approach to the stomach may be extremely difficult as the liver may occupy most of the surgical operative field [27,28,29]. This may not only result in an increased rate of post-BS complications but also in the suboptimal quality of surgery. A suboptimal surgery may be responsible for weight regain [30]. In addition, BS candidates often show preoperative MD [7,8].

Indeed, Ben-Porat et al. showed that the presence of pre-BS MD represents the strongest predictor of their presence in the post-BS period [31], suggesting that a specific supplemental program for each individual may consistently prevent postoperative micronutrient deficiencies. Moreover, Thibault et al. reported that in patients with obesity who are scheduled for BS, micronutrient status should be carefully monitored preoperatively [32], firstly, not only to optimize patients’ clinical conditions at the time of surgery but also to avoid an exacerbation of them after the surgery [6,33,34].

In this regard, we recently show that correcting MD before SG may be useful in preventing early postoperative MD [34]. However, despite all nutritional guidelines for BS suggesting a comprehensive preoperative macro- and micronutrient status assessment with the correction of any deficiency, in sufficient time before BS, MD are often left untreated in the BS setting [7], and more multicenter and randomized trials are necessary to determine whether the correction of MD before BS may have a positive impact in preventing MD after surgery in the long term. We acknowledge some limitations including the low number of patients and the lack of a control group. Furthermore, we are conscious that assessment of the liver volume by other techniques, such as magnetic resonance or computed tomography, could be more accurate. However, the obtained results on the left hepatic lobe assessment by ultrasonography appeared to be reliable and reproducible.

## 5. Conclusions

The lockdown determined by the SARS-CoV-2 pandemic has negatively affected the preoperative program of patients with obesity that are scheduled for BS, resulting in a postponement of surgery until the resumption of bariatric surgical activity. In view of further periods of lockdown with the consequent impossibility of following bariatric patients through classic outpatient activity, strengthening telematic strategies (e.g., telemedicine, video consultations, etc.) is essential for managing the preoperative nutrition of these patients more effectively and to avoid delays in surgical treatment. Further and larger randomized clinical trials are needed to confirm these preliminary data.

## Figures and Tables

**Table 1:** Composition of the supplement administered during the course of the study (Ketocompleat, MVMedical Solution, Serravalle, San Marino).

Element	Daily Value (40 g)	RDA *
Soy Protein	33.31 g	-
Vitamin A	0.483 mg	60.38%
Vitamin B1	0.496 mg	45.09%
Vitamin B2	0.833 mg	59.50%
Vitamin B3	8.33 mg	52.06%
Vitamin B5	1.28 mg	21.33%
Vitamin B6	0.92 mg	65.71%
Biotin	0.028 mg	56%
Vitamin B12	0.007 mg	280%
Folic Acid	0.222 mg	111%
Vitamin C	83.28 mg	104.10%
Vitamin D3	0.011 mg	222%
Vitamin E	12.65 mg	105.42%
Vitamin K1	11.1 mg	14.80%
Iron	5.5 mg	39.29%
Copper	0.08 mg	8%
Magnesium	39.9 mg	10.64%
Selenium	0.015 mg	27.27%
Manganese	0.19 mg	9.50%
Chromium	0.003 mg	7.50%
Calcium	116.76 mg	14.60%
Zinc	8.74 mg	87.40%
Inulin	1.38 g	-
Phaseolamin	1.11 g	-
Fructo-oligo-saccharides	1.11 g	-
Lactobacillus plantarum	2.24 mld	-
Lactobacillus acidophilus	2.2 mld	-
Lactobacillus rhamnosus	2.2 mld	-
Bifidobacterium longum	2.24 mld	-
Saccharomyces boulardi	4.48 mld	-

* Recommended Dietary Allowance.

**Table 2:** Characteristics of the study patients at baseline and after a 4-week course of preoperative KMED.

Clinical Characteristics	Baseline Mean ± SD	4-Week Follow Up Mean ± SD	*p*
Patients (M/F)	12/24	12/24	-
Body weight, kg (M/F)	143.6 ± 23.6	151 ± 16.1	0.115
BMI (kg/m^2^)	50.1 ± 5.9	52.8 ± 6.5	0.0692
Left hepatic lobe volume (cm^3^)	627 ± 85	631 ± 91	0.0848
Glucose (mg/dL)	115 ± 26.3	119 ± 23.9	0.502
Insulin (mU/L)	10.5 ± 5.9	10.9 ± 5.7	0.771
Iron (g/dL)	64.9 ± 12.7	66.5 ± 11.7	0.580
Hb (mcg/dL)	12.5 ± 5.9	12.9 ± 6.3	0.782
Creatinine (mg/dL)	0.86 ± 1.3	0.81 ± 1.2	0.866
Ketonemia (mmol/L)	0.05 ± 0.04	0.04 ± 0.02	0.184
Total Cholesterol (mg/dL)	224 ± 19.4	235 ± 21.5	0.0257
HDL (mg/dL)	51 ± 11.5	48 ± 11.9	0.280
LDL (mg/dL)	162 ± 27.9	167 ± 22.9	0.409
Total Cholesterol/HDL ratio	4.4 ± 2.9	4.9 ± 2.4	0.428
Triglycerides (mg/dL)	204 ± 35.4	221 ± 34.1	0.0416
GOT (U/L)	53 ± 14.1	56 ± 16.3	0.406
GPT (U/L)	39 ± 19.3	41 ± 18.6	0.656
GGT (U/L)	31 ± 19.6	33 ± 17.2	0.647
Uric Acid (mg/dL)	6.7 ± 1.2	6.9 ± 1.8	0.581
Urea/mg/dL)	31.4 ± 11.2	30.6 ± 13.2	0.782
GFR (mL/min)	95.4 ± 23.2	96.2 ± 26.3	0.891
† Vitamin B12 deficiency (M/F)	7/8	6/8	-
† Folate (M/F)	6/8	5/8	-
† Vitamin D deficiency (M/F)	10/12	10/12	-
† Iron deficiency (M/F)	4/8	2/8	-
† Zinc deficiency (M/F)	2/7	2/7	-
† Hypertension (M/F)	5/3	5/3	-
† Diabetes type 2 (M/F)	4/2	4/2	-
† Dyslipidemia (M/F)	4/4	4/4	-

## Data Availability

The data included in this manuscript derived from the University database. We are not authorized to share the data with third party organizations. However, the corresponding author is available to provide any explanation to the editor if requested.
